# Mr. Hennen's Case of Profuse Epistaxis

**Published:** 1804-06-01

**Authors:** John Hennen

**Affiliations:** Surgeon, third Division of Light Infantry. Strabane


### *To the Editors of the Medical and Physical Journal.*

GENTLEMEN,

THE following Case occurred to me at Lazaretto Barracks, Minorca, while Assistant Surgeon to the First Battalion of the Fortieth Regiment of Foot. As it exhibits a singular cause of profuse Epistaxis, I am induced, though at this distant period, to offer it for publication in your useful Journal.

I am, &c.

JOHN HENNEN,

Surgeon, third Division of Light Infantry.

*Strabane, April* 20, 1804.

---

WILLIAM STANNION, a private of the regiment, aged 25, was seized in the month of July, 1800, with violent pain in his forehead, in the site of the frontal sinusses; it was not constant, but when it did occur, which was generally at night, it usually lasted for three or four hours and was attended with violent hæmorrhage from the nose, which though frequently recurring, seldom lasted longer than ten minutes, and was constantly preceded by a sharp lancinating pain in the affected part. On the tenth day from the first attack, the common remedies of his comrades being insufficient to check the bleeding, he for the first time applied to me; I recognized him for an old patient, who had been under my care about two months before, while on the passage from England, in a very protracted case of typhus mitior, the most troublesome and obstinate symptom of which was pain in the same situation as that he now complained of, which continued long after every febrile symptom had dissappeared, and was attended with occasional amentia. Viewing his case as the effect of the intense heat acting on a weak habit, predisposed to congestion of blood in the head, a predisposition favoured by the pressure of his military accoutrements, I ordered him to remain as much as possible in the shade, to have his head frequently bathed in cold water, and I administered a gentle laxative, and introduced a dossil of lint dipped in solution of allum into the nostril of the right side, through which the blood flowed

 with with most violence. This treatment however was found to be insufficient, the hæmorrhage increasing, and great debility coming on; I therefore had recourse (on the fourth day after being consulted) to the injection of water artificially cooled by means of a solution of neutral salts. In a few minutes after its use, he felt something moving violently in the cavity of the nose, which gave him exquisite pain, and shortly after a substance resembling a clot of blood was discharged; he immediately uttered an exclamation of joy, saying he was relieved from most intense torture, and the hæmorrhage ceased altogether in about an hour. I, in the mean time, was occupied in examining the substance discharged, which, to my astonishment, as well as that of Mr. Bolton, the surgeon of the regiment, and my friend Mr. Woods, assistant of the second battalion, proved to be a leech, enveloped in coagulated blood; it was of the common size, and its motions were lively. I washed off the gore, and applied it on the hand of one of the hospital attendants; it readily fastened, and was not removed before it had produced the effects usually looked for from animals of its class, procured in the more common course of things; it survived this last effort twenty-four hours. From the history of this case, there cannot be a doubt of the animal discharged having been the cause of the hæmorrhage, and as little can be entertained of its having been in the cavity of the nose fourteen days at least previous to its removal. I shall not attempt to prove the exact time of the animal's residence in my patient's body, I would only observe that the barracks where he lay were at least three hundred yards from the sea, and that no fresh water, lake, or stream, was nearer than ten miles; nor indeed from every research that I could make, (which from my situation I confess was limited) could I ascertain that the leech is common in Minorca, though, according to Dr. Cleghorn, it is a native of that island. The patient never had a return of the epistaxis for eighteen months that I remained in the regiment; he recovered his health rapidly, and, except once that he had a venereal affection, at Ricasola, Malta, he never was afterwards in the sick report, nor was he unusually affected by the excessive heat of the island.

